# Evening exercise is associated with lower odds of visual field progression in Chinese patients with primary open angle glaucoma

**DOI:** 10.1186/s40662-020-0175-9

**Published:** 2020-03-01

**Authors:** Xiafei Pan, Kai Xu, Xin Wang, Guofu Chen, Huanhuan Cheng, Alice Jia Liu, Laurence Tang Hou, Lin Zhong, Jie Chen, Yuanbo Liang

**Affiliations:** 1 Department of Ophthalmology, Guangdong Provincial Key Laboratory of Malignant Tumor Epigenetics and Gene Regulation, Sun Yat-sen Memorial Hospital, Sun Yat-sen University, Guangzhou, China; 2 School of Ophthalmology and Optometry, Eye Hospital, Wenzhou Medical University, No. 270, Xue Yuan Xi Road, Wenzhou, 3250027 Zhejiang China; 3 Nanjing Sport Institute, No.8 Linggusi Road, Nanjing, Jiangsu China; 4 Glaucoma Institute, Wenzhou Medical University, Wenzhou, Zhejiang China; 5 Johns Hopkins University School of Medicine, Baltimore, MD 21205 USA

**Keywords:** Glaucoma, Exercise habits, Visual field progression

## Abstract

**Background:**

Exercise is widely known to lower intraocular pressure and increase ocular blood flow, which may be beneficial for glaucoma management. However, there are few studies that have reported on the relationship between exercise and glaucoma progression. The aim of our study was to investigate the exercise habits of those with primary open angle glaucoma (POAG) and its association with the progression of visual field (VF) loss.

**Methods:**

Daily physical activity (PA) was monitored by an accelerometer (ActiGraph wGT3x-BT) which patients wore for more than 10 h of being awake on their right wrists for 1 week.

**Results:**

Seventy-one non-progressive and 27 progressive patients were enrolled in the study. 24-h moderate to vigorous physical activity (MVPA) exercise showed that POAG patients had similar variation trends consisting of 3 wave peaks and 2 wave hollows. Minutes spent in MVPA was 19.89 ± 15.81 and 21.62 ± 15.10 during 07:00–09:00 h (*p* = 0.204), 15.40 ± 14.49 and 15.67 ± 12.43 during 15:00–17:00 h (*p* = 0.822) and 17.26 ± 21.11 and 11.42 ± 11.58 during 18:00–20:00 h (*p* = 0.001) in the non-progressive and progressive group, respectively. Univariate analysis indicated that 10 min of MVPA (18:00–20:00 h) [odds ratio, OR (95% CI) = 0.82 (0.73, 0.92)], average mean arterial pressure [OR (95% CI) = 0.96 (0.94, 0.98)], age [OR (95% CI) = 1.06 (1.03, 1.08)], male [OR (95% CI) = 0.67 (0.48, 0.96)], spherical equivalent [OR (95% CI) = 1.14 (1.07, 1.22)] and IOP-lowering medications [OR (95% CI) = 1.54 (1.16, 2.05)] were significantly correlated with having progressive VF damage. Multivariable analysis showed that 10 min of MVPA (18:00–20:00 h) [OR (95% CI) = 0.85 (0.75, 0.97)] was associated with progressive VF loss even after adjusting for other risk factors.

**Conclusions:**

Evening exercise may lower the odds of VF progression, suggesting that exercise habits possibly play an important role in glaucoma progression.

## Background

Glaucoma is a multifactorial optic neuropathy with an unclear pathogenesis which can result in irreversible visual field (VF) damage [1]. The number of glaucoma patients (40 to 80 years old) is estimated to be 76.0 million by 2020 worldwide, and projected to increase to 111.8 million by 2040 [2]. Currently, intraocular pressure (IOP) is the only modifiable risk factor for preventing glaucoma damage. However, patients diagnosed with normal tension glaucoma (NTG) and high-tension glaucoma (HTG) can still experience progressive VF loss even with a normal IOP. The Early Manifest Glaucoma Trial (EMGT) showed that 56% of untreated NTG patients progressed during the 6-year follow up [3]. In the Collaborative Normal-Tension Glaucoma Study (CNTGS), about one third of untreated subjects had localized progression within a three-year span, and up to 50% within 5 to 7 years [4]. Forty-five percent of open angle glaucoma patients demonstrated VF progression in the EMGT study [5]. In addition, large IOP reductions do not necessarily stop the progression of NTG. A comparison of the spontaneous untreated group and lowered-IOP group (decreased by 30% from baseline) in the CNTGS study found that 35% of control eyes and 12% of treated eyes showed optic disk progression or VF loss [6]. It is clear that non-IOP factors played an important role in the development of the disease since glaucomatous progression was only slowed, rather than halted in subjects with normal IOP levels.

Physical activity (PA) is an essential part of daily life, with PA guidelines in the US advising adults to set aside time for at least 150 min to 300 min of moderate intensity PA per week or 75 min to 150 min of vigorous intensity aerobic PA per week to stay healthy [7]. The health benefits of PA have been widely documented for both systemic and ocular diseases including boosting the immune system, improving sleep quality, reducing the incidence of cardiac-cerebral vascular events and is correlated with decreased anxiety and depression rates [8–12]. For eyes in particular, exercise can decrease the IOP [13] and decreasing IOP is associated with the magnitude of exercise [14]. Moreover, choroidal blood flow increases substantially in primary open angle glaucoma (POAG) patients with elevated exercise-induced blood pressure [15]. However, there is little conclusive evidence proving the effectiveness of exercise in mitigating glaucomatous VF loss, with many studies collecting patients’ physical activities through self-reported questionnaires [16, 17].

The objective of this study is to quantitatively monitor the daily PA of POAG patients and investigate the relationship between their exercise habits and progressive glaucomatous VF defect.

## Methods

The study was approved by the Institutional Review Board of Wenzhou Medical University (KYK [2018]20). Participants gave written informed consent and completed the study procedures between August 2017 and June 2018. The study followed the tenets of the Declaration of Helsinki.

### Study participants

Subjects were recruited from the Wenzhou Glaucoma Progression Study (WGPS), a longitudinal study exploring POAG patients’ glaucomatous clinical features and progression. POAG was defined as (1) open angles on gonioscopy, (2) glaucomatous optic disc changes, including neuroretinal rim narrowing, notching and retinal nerve fiber layer defects, (3) repeatable VF defects, (4) above changes in the absence of any other identifiable cause. Subjects were excluded if they were less than 18 years of age or they had any history of intraocular surgery (except uncomplicated cataract or glaucoma surgery).

All participants underwent a comprehensive ophthalmologic examination in the Clinical and Epidemiological Eye Research Center of the Eye Hospital of Wenzhou Medical University involving a questionnaire on the use of IOP-lowering medications and past medical history, gonioscopy, automated refraction (WAM-5500, Grand Seiko, Japan), Goldmann applanation tonometry (HAAG-STREIT 900 CM, Swiss), VF testing (Humphrey Field Analyzer IIi (HFA IIi, Carl Zeiss Meditec Inc., Dublin, CA), optical coherence tomography (OCT, Carl Zeiss Cirrus HD-OCT 4000, Germany), Lenstar (HAAG-STREIT LS900, Swiss), blood pressure (BP) measurements (Omron Automatic BP instrument (model HEM-7136, Omron Healthcare, Inc., IL) and other basic parameters. Patients were advised to follow up at the center every three to 6 months. Mean arterial pressure (MAP) was calculated as (1/3 systolic BP + 2/3 diastolic BP) while average MAP (aMAP) was the average MAP during all visits (from the baseline to the most recent visit). Mean IOP (mIOP) was obtained by averaging the IOP from all visits. PA was also measured during the follow-ups.

A total of 106 participants took part in the cross-sectional study. There were seven (6.60%) subjects with poor compliance and one (0.94%) subject whose device had technical issues. In the end, data from 98 patients were analyzed, including 27 patients in the progressive group and 71 patients in the non-progressive group. A valid day was defined as more than 10 h of awake wear time [18], and patients with less than six valid days were excluded from the study.

### Evaluation of physical activity

The accelerometer, ActiGraph wGT3x-BT (LLC, Pensacola, FL, USA), is an important tool that can accurately measure a person’s daily PA intensity and duration [19, 20]. Participants’ daily PA was recorded by the accelerometer (worn on the right wrist) for 1 week [21, 22]. Subjects were instructed on proper usage of the device and informed to take the device off when swimming or showering. Patients were also advised to wear the accelerometer for the entire day unless it was uncomfortable, or that it affected their sleep. Main measures of PA included calories burned per day, light PA (LPA) time per day, moderate PA (MPA) time per day, vigorous PA (VPA) time per day, very vigorous PA (VVPA) time per day, moderate to vigorous physical activity (MVPA) time per day and step counts. ActiLife software (version 6.13.3; ActiGraph, Pensacola, FL) was used to process the raw data and the values were presented as total daily and hourly counts per minute. Acceleration for x-, y-, and z-axes and vector magnitude (VM) were converted to 10 s epochs. Kcals was calculated by Freedson VM3 Combination (2011), kcals = S × [0.00097 × VM (x, y, z) + (0.08793 × BM)] - 5.01582, S = Duration of exercise (s)/60s, BM = Body Mass (kg) [23]. We used the validated Sasaki’s cut-point sets to estimate the amount of PA time: LPA, ≤2690 bouts; MPA, 2691~6166 bouts; VPA, 6167~9642 bouts; VVPA, ≥9643 bouts [24]. During this period, subjects were reminded to maintain their daily lifestyle and to not deliberately increase their total amount of exercise. Subjects were queried by phone call to verify the estimated wear time if any doubts about their compliance arose.

### Detection of visual field

Participants recruited from the WGPS needed to have at least 4 regular, reliable VF examinations (< 20% fixation losses and < 15% false-positive results). Humphrey VF testing was performed using the 24–2 Swedish Interactive Thresholding Algorithm (Standard) with stimulus size III after near refractive correction.

#### Definition: progressive VF defect

Progressive VF loss was defined as statistically significant deterioration (*p* < 0.05) on the pattern deviation change probability maps in the same three or more points with confirmation by at least two consecutive visits using the event-based glaucoma change probability (GCP) analysis of the Humphrey field analyzer in the Forum Glaucoma Workplace (Zeiss Carl-Forum 4.0) [25, 26].

Recruited glaucoma subjects were divided into the progressive and non-progressive group depending on the nature of their disease. Daily PA was compared between these two groups. Patients were classified as a progressor if they had at least one eye with progressive VF loss. Data for both eyes were obtained, and for the non-progressive group, the worse eye was chosen. For patients with two progressive eyes, the worse progressive eye was chosen, and for patients with only one progressive eye, the progressive eye was selected. The average data of all visits, such as IOP and MAP were used in the study. The presenting visual acuity at the time of PA assessment was used and data involving other parameters were from the baseline.

## Statistical methods

Statistics were performed using SPSS (version 21.0). A *p* value of less than 0.05 was considered statistically significant. Demographics, ocular characteristics, and PA are presented using mean and standard deviation and compared between the non-progressive and progressive group using an independent sample t-test for normally-distributed continuous variables, the Mann-Whitney U test for non-normally distributed continuous variables, and a Chi-squared test for categorical variables. Logistic regression was used to explore the association of binary dependent variable “progression of glaucoma” with continuous or categorical independent variables.

## Results

Seventy-one patients with non-progressive VF loss (61.45 ± 12.99 years, 53.52% male) and 27 patients with progressive VF loss (67.22 ± 8.93 years, 44.44% male) who had no less than 6 days of valid accelerometer data were enrolled from the WGPS. POAG patients with and without progressive VF damage were similar in gender, body-mass index, mIOP, aMAP, visual field MD, retinal nerve fiber layer, presenting visual acuity, spherical equivalent, axial lengths, central corneal thickness, IOP-lowering medications, self-reported hypertension, self-reported diabetes, total days of PA measurement, follow-up period and number of follow-ups (*p* > 0.05 for all), but differed in age (*p* = 0.023) (Table 1).

**Table 1:** Demographics and ocular characteristics of non-progressive and progressive patients

Parameter	Non-progressive	Progressive group (n = 27)	*p* value
	group (*n* = 71)		
Gender (% male)	38 (53.52%)	12 (44.44%)	0.422^a^
Age (yrs)	61.45 ± 12.99	67.22 ± 8.93	**0.023**^b^
BMI (kg/m^2^)	24.32 ± 3.09	23.27 ± 3.39	0.143^c^
mIOP (mmHg)	15.30 ± 2.68	15.39 ± 3.71	0.900^c^
aMAP (mmHg)	94.00 ± 8.43	90.81 ± 9.28	0.107^c^
Visual field MD (dB)	−7.44 ± 5.24	−6.71 ± 5.98	0.233^b^
RNFL (μm)	76.54 ± 12.93	76.56 ± 14.16	0.995^c^
Presenting visual acuity (logMAR)	0.23 ± 0.20	0.28 ± 0.20	0.222^b^
SE (D)	−0.89 ± 3.14	0.06 ± 2.74	0.117^b^
AL (mm)	24.19 ± 1.72	23.86 ± 1.05	0.520^b^
CCT (μm)	539.07 ± 30.18	540.89 ± 28.84	0.788^c^
No. of visual field (MD)			0.744 ^a^
MD ≥ −12 dB [n (%)]	67 (94.40%)	25 (92.60%)	
MD < −12 dB [n (%)]	4 (5.60%)	2 (7.40%)	
IOP-lowering medications			0.228^a^
Non-medications [n (%)]	52 (73.20%)	17 (63.00%)	
One medication [n (%)]	17 (23.90%)	9 (33.30%)	
Two medications [n (%)]	2 (2.80%)	0 (0.00%)	
Three medications [n (%)]	0 (0.00%)	1 (3.70%)	
Self-reported hypertension [n (%)]	21 (29.6%)	6 (22.2%)	0.467^a^
Self-reported diabetes [n (%)]	30 (42.3%)	8 (29.6%)	0.252^a^
Total days of PA measurement	6.82 ± 0.39	6.70 ± 0.47	0.225^b^
Follow-up period (months)	41 ± 10.89	31.11 ± 10.32	**< 0.001**^b^
Number of Follow-ups (n)	11.20 ± 3.97	6.85 ± 2.58	**< 0.001**^b^

*BMI* = body-mass index; *mIOP* = mean intraocular pressure during all visits; *MD* = mean deviation in the baseline; *RNFL* = retinal nerve fiber layer detected by OCT; *aMAP* = average mean arterial pressure during all visits; *logMAR* = logarithm of the minimum angle of resolution presenting visual acuity measured at the time of PA assessment; *SE* = spherical equivalent; *AL* = axial lengths; *CCT* = central corneal thickness; *No. of visual field (MD)*, visual field MD was measured at the time of PA assessment, MD ≥ −12 dB, mild to moderate VF damage; MD < − 12 dB, serious VF damage; *PA* = physical activity. Data are represented as mean ± SD

^a^Chi-squared test, ^b^Mann-Whitney U test, ^c^Independent Sample T test. Bold values indicate statistical significance with alpha set at 0.05

Median calories burned per day was 3563.44 ± 1832.82 kcal and 2909.29 ± 1250.14 kcal in the non-progressive and progressive group, respectively (*p* = 0.111). A comparison of POAG patients’ total PA duration for all exercise levels (light, moderate, vigorous, very vigorous and MVPA) revealed no significant differences between the two groups (*p* > 0.05 for all). There was also no significant difference for total step count between the two groups (*p* = 0.617) (Table 2).

**Table 2:** Difference in physical activity time between non-progressive and progressive patients

Parameter	Non-progressive group (*n* = 71)	Progressive group (*n* = 27)	*p* value
Kcals (per day)	3563.44 ± 1832.82	2909.29 ± 1250.14	0.111^a^
Light PA (min/day)	1360.27 ± 54.31	1365.03 ± 32.62	0.611^a^
Moderate PA (min/day)	80. 82 ± 41.20	75.28 ± 30.33	0.527^b^
Vigorous PA (min/day)	4.06 ± 8.87	1.43 ± 1.32	0.469^a^
Very vigorous PA (min/day)	0.28 ± 0.47	0.20 ± 0.26	0.975^a^
MVPA (min/day)	85.16 ± 45.67	76.91 ± 31.24	0.311^b^
Steps (per day)	9738.42 ± 4248.72	9379.48 ± 2632.88	0.617^b^

*Kcal* = calories burned after exercise; *PA* = physical activity; *MVPA* = moderate to vigorous physical activity. Data are represented as mean ± SD. ^a^Mann-Whitney U test. ^b^Independent Sample T test

**Fig. 1:**
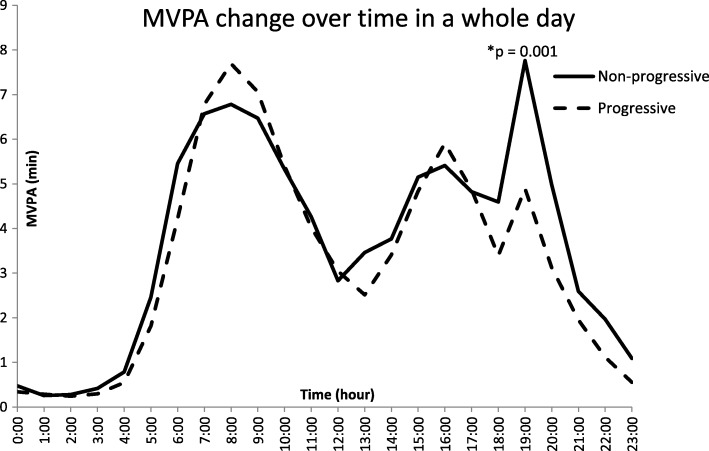
24-h variation of MVPA time shown (3 wave peaks and 2 wave hollows) for the non-progressive and progressive groups. The MVPA time have significant difference between those two groups during 18:00–20:00 pm, but have no statistical difference during 07:00–09:00 am and 15:00–17:00 pm

The line graph illustrates the variation of MVPA time subjects spent over a whole day. MVPA fluctuations for the non-progressive and progressive groups had similar trends in variation (3 wave peaks and 2 wave hollows). The average number of minutes patients spent in MVPA during 07:00–09:00 h was 19.89 ± 15.81 and 21.62 ± 15.10 (*p* = 0.204), 15.40 ± 14.49 and 15.67 ± 12.43 during 15:00–17:00 h (*p* = 0.822), and 17.26 ± 21.11 and 11.42 ± 11.58 during 18:00–20:00 h (*p* = 0.001) in the non-progressive and progressive group, respectively (Fig. 1).

To investigate other possible factors influencing progressive VF loss of POAG patients, we conducted a binary logistic regression (univariate and multivariable analysis). Univariate analysis indicated that 10 min of MVPA (18:00–20:00 h) [Odds ratio, OR (95% CI) = 0.82 (0.73, 0.92)], average mean arterial pressure (aMAP) [OR (95% CI) = 0.96 (0.94, 0.98)], age [OR (95% CI) = 1.06 (1.03, 1.08)], male [OR (95% CI) = 0.67 (0.48, 0.96)], spherical equivalent (SE) [OR (95% CI) = 1.14 (1.07, 1.22)] and IOP-lowering medications [OR (95% CI) = 1.54 (1.16, 2.05)] were significantly correlated with having progressive VF damage. Multivariable analysis showed that the following features were associated with progressive VF defect: 10 min spent in MVPA [OR (95% CI) = 0.85 (0.75, 0.97)], aMAP [OR (95% CI) = 0.95 (0.93, 0.97)], age [OR (95% CI) =1.08 (1.05, 1.11)], SE [OR (95% CI) = 1.11 (1.02, 1.20)] and lowering-IOP medications [OR (95% CI) = 2.95 (2.02, 4.31)] (Table 3).

**Table 3:** Univariate and multivariable odds ratio and 95% confidence intervals for VF progression of POAG patients

Parameter	Univariate				Multivariable			
	OR	95% CI		*p* value	OR	95% CI		*p* value
		lower	upper			lower	upper	
MVPA (per 10 mins) (18:00–20:00 pm)	0.82	0.73	0.92	**0.001**	0.85	0.75	0.97	**0.016**
MD (dB)	1.03	1.00	1.06	0.074				
RNFL (μm)	1.00	0.99	1.01	0.854				
aMAP (mmHg)	0.96	0.94	0.98	**< 0.001**	0.95	0.93	0.97	**< 0.001**
mIOP (mmHg)	1.02	0.96	1.08	0. 511				
Age (yrs)	1.06	1.03	1.08	**< 0.001**	1.08	1.05	1.11	**< 0.001**
Gender (male)	0.67	0.48	0.96	**0.027**	1.15	0.75	1.75	0.520
CCT (μm)	1.00	1.00	1.01	0.719				
SE (D)	1.14	1.07	1.22	**< 0.001**	1.11	1.02	1.20	**0.014**
IOP-lowering medications	1.54	1.16	2.05	**0.003**	2.95	2.02	4.31	**< 0.001**

*CI* = confidence interval; *MVPA* = moderate to vigorous physical activity time per ten minutes when patients exercise between 18:00 to 20:00 pm; *MD* = mean deviation at the baseline; *RNFL* = average retinal nerve fiber layer detected by OCT at the baseline; *aMAP* = average mean arterial pressure during all visits, calculated by (systolic blood pressure + 2 × diastolic blood pressure) ÷ 3; *mIOP* = mean intraocular pressure during all visits; *SE* = spherical equivalent; *CCT* = central corneal thickness; *IOP-lowering medications*, the number of IOP-lowering medications. Bold values indicate statistical significance with alpha set at 0.05

## Discussion

This study objectively monitored the 24-h PA of POAG patients to provide a solid portrayal of their daily PA lifestyle. Adjusted OR for MVPA time per 10 min (18:00–20:00 h) was 0.85 (95% CI: 0.75–0.97) for subjects with progressive VF defect in comparison to those with non-progressive VF damage, indicating an increase of time spent in MVPA by 10 min decreases the odds of progressive VF defect by 15%. However, daily PA time of different intensity had no statistical association with progressive glaucomatous VF loss. Lee and his team [21] investigated 141 suspects or manifest glaucoma patients and found that increased PA such as walking, MVPA, and non-sedentary activity were associated with slower rates of VF loss. This discrepancy may have arisen from differences in subject characteristics (POAG vs. suspects and manifest glaucoma), measurement of VF progression (progressor vs. the rate of VF loss) and enrollment criteria (without intraocular surgery vs. conditional surgery).

The levels of VF damage may affect patients’ daily physical activities. Ramulu [22] monitored 83 glaucoma subjects and 58 controls for their daily minutes of MVPA and steps per day, finding that daily PA time was impacted by glaucomatous VF loss. In our study, the chosen eye (worse or progressive eye) VF mean deviations were − 6.22 ± 6.09 dB in the non-progressive group and − 7.73 ± 5.45 dB in the progressive group (*p* = 0.084) at the time of PA assessment. In addition, the percentage of people with mild to moderate VF defect was 94.40 and 92.60% in these two groups (*p* = 0.744). Overall, no significant difference in presenting visual acuity was found between the two groups, reducing the possibility that progressors exercise less in the evening due to the severe VF loss.

This is the first study showing a correlation between evening exercise (18:00–20:00 h) and VF loss progression in glaucoma. Circadian rhythms and cyclical physiological changes over a 24-h period may play an important role in the findings as the choice of what time of day to exercise heavily influences physiological variables. Many biological functions have been shown to be time-of-day dependent as their physiological levels fluctuate throughout the day. Ammar A [27] summarized the effects of time-of-day on many physiological functions and how biological parameters adjust in response to increased PA of healthy subjects. In the early evening, individuals have a higher rate of oxidative stress, white blood cell count, homocysteine and muscle damage markers, and a more efficient antioxidant activity than in the early morning. Trabelsi K [28] explored the diurnal variation (08:00 h, 14:00 h and 18:00 h) of the same hematological parameters and found that total white blood cell count (WBC) (*p* < 0.01) and neutrophils count (NE) (p < 0.01) have higher resting values in the early evening than in the morning. Previous research has also shown that the impact of repeated sprinting exercises on hematocytes [WBC, NE, lymphocytes (LY) and monocytes (MO)] being dependent on the time-of-day, with greater values obtained in the early evening [29]. Additionally, the effect of time-of-day on many physiological functions can change in response to PA. Studies demonstrate that in the evening, aerobic exercise’s time to exhaustion, body temperature, peak oxygen consumption and aerobic system response, are higher in comparison to the same measures in the morning [27, 30, 31]. Evening exercise is better than morning exercise for fat oxidation levels and energy expenditure (EE) as EE and oxygen uptake are higher in the evening [32, 33]. Nikkhah A [34] also demonstrated that early night exercise can decrease cellular irresponsiveness to insulin, particularly for those overweight or obese. Metabolic levels not only depend on the time-of-day, but also change in response to PA. Therefore, an individual’s metabolic levels vary depending on when he or she chooses to exercise. However, the underlying mechanism responsible for the relationship between evening exercise and glaucomatous VF progression remains unclear.

Furthermore, it is widely believed that elevated IOP, vascular dysregulation, and perfusion deficit may lead to glaucoma progression [35, 36]. Elevated IOP is the only modifiable risk factor for most glaucoma subtypes, including POAG. Decreased ocular perfusion and vascular dysregulation has also been implicated as a major risk factor for glaucoma [37, 38]. Past research has shown that dynamic exercise significantly reduces IOP and increases ocular blood flow. Natsis K [39] found that aerobic exercise can help reduce IOP even when patients are already on various antihypertensive drugs. Hayashi N [40] reported that increasing exercise intensity induces rising retina and choroidal blood flow. Portmann N [15] explored the response of submacular choroid blood flow (ChBF) to isometric exercise and observed that POAG patients have a smaller active regulatory capacity and a larger increase in ChBF after exercising in comparison with their healthy counterparts, indicating that ocular blood flow in POAG patients is more sensitive to exercise. Taken together, exercise not only decreases IOP, but also increases ocular perfusion (ChBF, ocular perfusion pressure and ocular blood pressure), which may explain why more PA is associated with lower risk of glaucomatous progressive VF damage.

Much like other patients with chronic diseases, glaucoma patients are more likely to have depression with a reported prevalence of 10.9% in comparison with those without glaucoma (6.9%) [41]. Literature has shown that exercise reduces the incidence of depression or anxiety [8, 42, 43]. For example, Babyak M [44] investigated 156 adult volunteers with major depressive disorder for 10 months, and found that increasing exercise time lowers depression relapse rates in comparison to that of the medication group. Apart from alleviating depression, exercise can also improve sleep quality, reduce morbidity of cardiovascular diseases, boost the immune system, and even promote neuronal plasticity [8–12, 45], and thus have comprehensive benefits not only for the eyes, but the entire body.

An advantage of our study includes using a widely recognized accelerometer to.

measure the 24-h PA of patients to provide objective, comprehensive results. Additionally, our study is the first to discuss the relationship of exercise habits and glaucomatous VF progressive defect, finding that more evening exercise was associated with lower odds of VF progression. During the follow-up visits, we collected data on mean IOP and MAP and adjusted them in the multivariable analysis. Furthermore, the diagnosis of POAG was performed clinically, which was more accurate than population-based designs [16, 17]. However, there are several limitations in our work: Firstly, our study is an observational study, so we cannot conclude that there is a causal relationship between evening exercise and progressive VF defect. Secondly, the device is not waterproof, and we did not monitor swimming PA time, which may have resulted in a lesser amount of recorded PA time. Anticipating this, we asked all our subjects if they swim regularly and found that 93 (94.9%) never swim, one (1.0%) swims regularly in the summertime and four (4.1%) occasionally swim, indicating this to be a minor flaw. Thirdly, using the monitor to measure the daily PA of the elderly with visual impairment may have some deviations than that in the other elderly. However, to our knowledge, no validity studies exist on tools that measure PA in patients with ocular disorders. Valid and specific tools need to be designed in future studies when measuring daily PA for the elderly with visual impairment. Lastly, age was not matched in the progressive and non-progressive group and there was no control group without POAG. Although the age factor was adjusted in the multivariate analysis, a health control group could provide a well-balanced comparison between the groups.

## Conclusions

In conclusion, our study found that more evening exercise (MVPA) was associated with lower odds of progressive VF damage in patients with POAG. Our data suggest that exercise habits may play an important role in glaucoma progression, but a prospective, interventional study is needed to confirm our findings.

## Data Availability

The datasets used and/or analyzed during the current study are available from the corresponding author on reasonable request.

## Abbreviations

AL: Axial lengths
BMI: Body-mass index
CCT: Central corneal thickness
ChBF: Choroid blood flow
CNTGS: Collaborative Normal-Tension Glaucoma Study
EE: Energy expenditure
EMGT: Early Manifest Glaucoma Trial
HTG: High-tension glaucoma
IOP: Intraocular pressure
IQR: Interquartile Range
LY: Lymphocytes
MAP: Mean arterial pressure
MO: Monocytes
MVPA: Moderate to vigorous physical activity
NE: Neutrophils count
NTG: Normal tension glaucoma
OR: Odds ratio
PA: Physical activity
POAG: Primary open angle glaucoma
RNFL: Retinal nerve fiber layer
SE: Spherical equivalent
VF: Visual field
WBC: White blood cell
WGPS: Wenzhou Glaucoma Progression Study
